# MicroRNAs Targeting Oncogenes Are Down-Regulated in Pancreatic Malignant Transformation from Benign Tumors

**DOI:** 10.1371/journal.pone.0032068

**Published:** 2012-02-22

**Authors:** Long R. Jiao, Adam E. Frampton, Jimmy Jacob, Loredana Pellegrino, Jonathan Krell, Georgios Giamas, Nicole Tsim, Panagiotis Vlavianos, Patrizia Cohen, Raida Ahmad, Andreas Keller, Nagy A. Habib, Justin Stebbing, Leandro Castellano

**Affiliations:** 1 Hepato-Pancreato-Biliary Surgical Unit, Department of Surgery and Cancer, Imperial College, Hammersmith Hospital, London, UK; 2 Division of Oncology, Department of Surgery and Cancer, MRC Cyclotron Building, Imperial College, Hammersmith Hospital, London, UK; 3 Department of Gastroenterology, Imperial College NHS Trust, Hammersmith Hospital, London, UK; 4 Department of Histopathology, Imperial College NHS Trust, Hammersmith Hospital, London, UK; 5 Department of Human Genetics, Saarland University, Homburg, Saar, Germany; University of Frankfurt - University Hospital Frankfurt, Germany

## Abstract

**Background:**

MicroRNA (miRNA) expression profiles have been described in pancreatic ductal adenocarcinoma (PDAC), but these have not been compared with pre-malignant pancreatic tumors. We wished to compare the miRNA expression signatures in pancreatic benign cystic tumors (BCT) of low and high malignant potential with PDAC, in order to identify miRNAs deregulated during PDAC development. The mechanistic consequences of miRNA dysregulation were further evaluated.

**Methods:**

Tissue samples were obtained at a tertiary pancreatic unit from individuals with BCT and PDAC. MiRNA profiling was performed using a custom microarray and results were validated using RT-qPCR prior to evaluation of miRNA targets.

**Results:**

Widespread miRNA down-regulation was observed in PDAC compared to low malignant potential BCT. We show that amongst those miRNAs down-regulated, *miR-16, miR-126* and *let-7d* regulate known PDAC oncogenes (targeting BCL2, CRK and KRAS respectively). Notably, *miR-126* also directly targets the KRAS transcript at a “seedless” binding site within its 3′UTR. In clinical specimens, *miR-126* was strongly down-regulated in PDAC tissues, with an associated elevation in KRAS and CRK proteins. Furthermore, *miR-21*, a known oncogenic miRNA in pancreatic and other cancers, was not elevated in PDAC compared to serous microcystic adenoma (SMCA), but in both groups it was up-regulated compared to normal pancreas, implicating early up-regulation during malignant change.

**Conclusions:**

Expression profiling revealed 21 miRNAs down-regulated in PDAC compared to SMCA, the most benign lesion that rarely progresses to invasive carcinoma. It appears that *miR-21* up-regulation is an early event in the transformation from normal pancreatic tissue. MiRNA expression has the potential to distinguish PDAC from normal pancreas and BCT. Mechanistically the down-regulation of *miR-16, miR-126* and *let-*7d promotes PDAC transformation by post-transcriptional up-regulation of crucial PDAC oncogenes. We show that *miR-126* is able to directly target KRAS; re-expression has the potential as a therapeutic strategy against PDAC and other KRAS-driven cancers.

## Introduction

Pancreatic cancer is the 4^th^ commonest cause of cancer-related death accounting for 33,000 deaths per year in the US [1], [2], [3] and at least 6,000 deaths per year in the UK [4]. Currently surgical resection remains the only treatment associated with the potential for cure [5]. However, most patients have locally advanced or metastatic disease at presentation and are therefore not surgical candidates [3], [6]; the actual resection rate is less than 10% [7]. Routine imaging techniques such as computed tomography (CT) or magnetic resonance imaging (MRI) are not sensitive enough to detect pancreatic cancer at an early stage [2]. In addition, patients continue to be diagnosed with advanced disease because currently there are no tumor markers that allow reliable screening at a potentially curable stage.

Cystic lesions of the pancreas can be either inflammatory or neoplastic [8], [9]. The epithelial benign cystic tumors (BCT) of the pancreas have the potential to transform into invasive pancreatic ductal adenocarcinoma (PDAC) (Figure S1). Clinical differentiation between low and high-risk pre-malignant BCT can be difficult and the consequences of missing the chance for a curative procedure in patients who are suitable for pancreatic surgical resection can be devastating [8]. BCT are divided into non-mucinous and mucinous variants: serous microcystic adenomas (SMCA), which are non-mucinous tumors, have a very low-malignant potential (<2%) and very rarely progress to PDAC [10]; intraductal papillary mucinous neoplasms (IPMN) are mucinous tumors that are connected to the native pancreatic ducts (main or side-branch) [11]; whilst the mucinous cystic neoplasms (MCN) are separate from the ductal system [11], [12]. Main branch IPMN lesions carry the highest malignant potential, ranging between 57 to 92% and side-branch IPMN between 6 to 46% [12], [13]. MCNs have a high-malignant potential ranging from 6 to 36% [14], [15]. Out of the BCT, the most often encountered are the SMCA (32%–39%), MCNs (10%–45%), and IPMNs (21%–33%) [16]. The latter have more potential to give rise to *in situ* or invasive PDAC, via an adenoma-carcinoma sequence [3], [5], [14]. Invasive malignancy arising on the background of an IPMN is termed Carcinoma-Ex-IPMN (CEI) and is more common in main pancreatic duct IPMN [12], [15], [17]. A correct preoperative diagnosis and evaluation of pancreatic BCT is crucial for clinical decision-making to sieve out those tumors that are already malignant or have a high-risk of malignant potential for which urgent surgical intervention is required [17].

MiRNAs are a recently recognized class of non-coding short RNAs from 17 to 25 nucleotides in length that play a role in post-transcriptional gene regulation [18]. Expression profiles of human miRNAs have demonstrated that many miRNAs are deregulated in cancer and these profiles will help further establish molecular diagnosis, prognosis and therapy. Several studies have demonstrated a different miRNA expression profile in PDAC compared to normal tissues [2], [19], [20]. However, the profiles of miRNA production in PDAC precursor lesions remain largely unknown.

In this report, miRNA expression signatures in low and high-risk pre-malignant pancreatic BCT were investigated. Furthermore, the role of oncogene targeting miRNAs in the regulation of malignant transformation from BCT was assessed and KRAS was identified as a direct target of *miR-126*. Ultimately, identification of miRNA markers for the clinical differentiation of these pre-malignant BCT would allow for early surgical resection to improve outcomes.

## Methods

### Tissue samples

Analysis of miRNAs in historical stored formalin-fixed paraffin embedded (FFPE) and fresh surgical specimens was approved by a UK national research ethics committee (London; 09/H0722/77) and by Imperial College Healthcare NHS Trust. Following written informed consent, specimens were obtained from 58 individuals who underwent pancreatic resection for a cystic tumor or known PDAC between May 1999 and November 2010 at the Hammersmith Hospital, London, UK. During this period, 4 FFPE and 9 fresh samples of normal pancreas were also collected from pancreatic resection following trauma. After macroscopic examination, 10 µm thick sections were obtained from the paraffin blocks for the FFPE tumor samples (n = 43) as in previous studies [19], [21], [22]. For the FFPE microarray there were: SMCA (n = 7), MCN (n = 6), IPMN (n = 7), CEI (n = 9) and PDAC (n = 14). Our histopathologist removed any adjacent normal acinar or adipose tissue with a scalpel. In addition, several sections (3 to 5) were taken from each block in order to ensure that a representative sample was obtained. Fresh tissue samples (n = 24; normal pancreas n = 9, PDAC n = 15) collected at surgery were immediately placed in RNA*Later* RNA Stabilization Reagent solution (Qiagen, Hilden, Germany) and stored at room temperature for 2–3 hours before being frozen at −80°C. The immunohistochemical analysis was performed on FFPE samples: normal pancreas n = 12, PDAC n = 12 and SMCA n = 12 (an additional 5 cases of SMCA were available at this time). Further detailed clinicopathological information about the patients is provided in Table S1.

### Cell culture and transfection

PANC-1 and MIA PaCa-2 pancreatic cells were purchased from the European Collection of Cell Cultures (ECACC). Both were maintained in 50% DMEM and 50% RPMI supplemented with 10% FCS, 1% penicillin/streptomycin, and 1% glutamine. When the cells were ready for transfection, they were plated in 6 well plate the day before and then transfected with precursor miRNA (pre-miR) or miRNA inhibitor (anti-miR) (Applied Biosystems, Cheshire, UK) for 48 hours using HiPerFect Transfection Reagent (Qiagen, Hilden, Germany) before lysis, RNA and protein extraction.

### RNA Isolation

FFPE samples were deparaffinized with xylene and total RNA was collected using the miRNeasy Mini Kit (Qiagen, Hilden, Germany) according to the manufacturer's instructions. Fresh tissue stored in RNA*Later* was crushed in liquid nitrogen and subsequent powder lysed in Trizol Reagent (Invitrogen, Paisley, UK), followed by RNA isolation according to the manufacturer's instructions.

### miRNA Microarray

The microarray we used is applicable and has been validated for FFPE samples [23]. Total RNA was extracted (as mentioned previously) and the samples were analyzed with the Geniom Realtime Analyzer (GRTA) using the Geniom Biochip MPEA *Homo sapiens* (both by febit biomed gmbh, Germany).

The probes on the biochip are designed as the reverse complements of all major mature human miRNAs (866 miRNAs) as published in the Sanger miRBase version 13.0 (March 2009) [24], [25]. The probes are synthesized with 7 intra-array replicates for each miRNA to increase the statistical confidence and to compensate for potential positional effects. This microarray combined with the fully automated GRTA platform allows for measuring miRNA signatures and ensures a high degree of reproducibility [26]. Samples were labeled by microfluidic-based enzymatic on-chip labeling of miRNAs (MPEA) [27]. Following hybridization for 16 hours at 42°C, the biochip was washed automatically and a program for signal enhancement was processed with the GRTA. Resulting detection pictures were evaluated using the Geniom Wizard Software (febit biomed gmbh, Germany).

We have deposited the raw data at GEO under accession number GSE29352, we can confirm all details are MIAME compliant.

### RT-qPCR

A selection of miRNAs were chosen for validation based on statistical significant high levels of logarithmized fold change seen on the microarray, as well as their known potential roles in tumorigenesis. Extracted total RNA was used to perform RT-qPCR using Taqman mature miRNA primers and probes (Applied Biosystems, Cheshire, UK). Briefly, RNA was reverse transcribed followed by qPCR on a 7900 HT Fast Real-Time PCR System (both by Applied Biosystems, Cheshire, UK). Duplicate samples and endogenous controls (U6, U47 and *miR-191*) were used throughout. Expression levels of each miRNA were evaluated using the comparative threshold cycle (Ct) method as normalized to a control (2 ^−ΔCt^). The relative expression levels of each miRNA were calculated between tissue types.

For gene expression analysis, total RNA was reverse transcribed using Superscript III Reverse Transcriptase (Invitrogen, Paisley, UK) and cDNA transcripts were amplified by qPCR using SYBR Green (Applied Biosystems, Cheshire, UK). Triplicate samples were used and levels were normalized to GAPDH using primers described in Castellano *et al* [18]. KRAS primer sequences were from Kent *et al* [28].

### Luciferase Reporter Assay

For KRAS 3′UTRs reporter construction, complementary oligonucleotides (Sigma Aldrich Ltd., Dorset, UK) containing the *miR-126* recognition elements (MRE) plus 10 nucleotides on each side were annealed and successively cloned into the Mlu1 and HindIII sites of the multiple cloning site (MCS) of pMIR-REPORT *Firefly* Luciferase vector (Applied Biosystems, Cheshire, UK). KRAS 3′-UTR containing two wild-type (named KRAS_A_WT and KRAS_B_WT) and two mutated (named KRAS_A_MUT and KRAS_B_MUT) *miR-126* binding sites were used to produce the constructs. The sequences of all primers used for plasmid construction are reported in Table S2.

MIA PaCa-2 cells were seeded onto 24-well plates (10×10^5^ cells per well) the day before transfections were performed. Cells (80% confluent) were co-transfected with pRL-TK luciferase reporters (50 ng/well), pMIR-REPORT firefly luciferase (150 ng/well), and pre-miR-126 (100 nmol/L) using Lipofectamine 2000 (Invitrogen, Paisley, UK). After 48 hours the cells were lysed using a passive lysis buffer (Promega, Southampton, UK) and the firefly and *Renilla* luciferase luminescence signals were measured using the Dual-Glo Luciferase Assay System (Promega, Southampton, UK).

### Western Blotting

Whole cell lysates were prepared in Nonidet P-40 lysis buffer [50 mM Tris/HCl, pH 8.0, 150 mM NaCl, 10% (vol/vol) glycerol, 1% Nonidet P-40, 5 mM DTT (DTT), 1 mM EDTA, 1 mM EGTA, 50 µM leupeptin, and 30 µg/mL aprotinin]. Lysates were subjected to SDS/PAGE and blotted on a Hybond C super nitrocellulose membrane (GE Healthcare, Bucks, UK). The intensity of bands was quantified using Image J software (National Institutes of Health). We used BCL2 (ab692) (Abcam Plc., Cambridge, UK), CRK (610035) (BD Ltd., Oxford, UK), KRAS (sc-30) and GAPDH (sc-137179) (Santa-Cruz Biotechnology Inc., Santa-Cruz, USA) monoclonal mouse antibodies.

### Immunohistochemistry

Sections (4 µm) from FFPE blocks were prepared for immunohistochemical examination. After deparaffinisation and rehydration, antigen retrieval was performed by boiling in 10 mmol/l of citrate buffer (pH 6.0) for 10 min. After inhibition of endogenous peroxidase activity for 30 min with methanol containing 0.3% H_2_O_2_, the sections were blocked with 2% BSA in PBS for 30 min and incubated with antibodies against CRK (as before). The immune complex was visualised with the Dako REAL EnVision Detection System, Peroxidase/DAB, Rabbit/Mouse (Dako, Cambridgeshire, UK), according to the manufacturer's procedure. The nuclei were counterstained with hematoxylin. Representative photographs were taken and two pathologists (R.A. and P.C.) scored the slides for protein expression.

### Statistical analysis

The miRNA microarray aimed to detect differential expression between tissue types. The mean expression values for each miRNA on the microarray were first background subtracted and normalized before analysis. Global background subtraction corrects for several experimental factors that may cause a systematic spatial variability on a microarray. Following this, the 7 replicate intensity values of each miRNA were summarized by their median value. Quantile normalization was then performed across all the different arrays [29]. These microarray data are presented as the median relative miRNA expression levels observed and the median logarithmized fold changes between tissue types.

A hierarchical clustering heatmap was created using the 35 miRNAs with the highest variability in order to separate the data graphically. This was done because if all the miRNAs were used then there would be no reliable image, since most are contributing more background noise than signal. To detect whether partitioning was significant, a 3×3 contingency table consisting of the 3 main groups of tissue type (PDAC, CEI and BCT), was analysed using Fisher's Exact test [30]. A *P*<0.05 was considered a significant clustering result.

Limma is a test for differential expression analysis of data arising from microarray experiments. Empirical Bayes and other methods are used to borrow information across genes, making the analyses ideal for experiments with a small number of arrays [31], [32]. The resulting *P*-values were adjusted for multiple testing by the Benjamini-Hochberg method [33], [34]. A log fold change for a deregulated miRNA with a limma adjusted *P*<0.05 was considered statistically significant. Tables S3, S4, S5, and S6 demonstrate the microarray results for the 30 most deregulated probes (detected by highest absolute value of logarithmized fold changes) in each tissue comparison.

The differential miRNA expression between tissues for all RT-qPCR and Western blotting data was analyzed using the parametric *t*-test (unpaired, 1-tailed for validation of the FFPE samples and unpaired, 2-tailed for fresh tissue samples) with Graphpad Prism 4.0 (Graphpad Software Inc, San Diego, California). The immunohistochemistry data was analyzed using a 3×3 contingency table and the Fisher's Exact test (2-tailed). Where required, the *P*-values were adjusted for multiple testing with the Bonferroni correction.

## Results

### Microarray expression profiles reveal general miRNA down-regulation in PDAC compared to low malignant potential BCT

In order to distinguish the various types of pancreatic tumor, miRNA expression profiling was performed using total RNA derived from FFPE tissues of low and high malignant potential BCT and ductal adenocarcinoma (CEI and PDAC). It has already been described that PDAC is mainly characterized by miRNA up-regulation. Bloomston *et al*. identified 30 miRNAs up-regulated and 3 down-regulated in PDAC compared to normal pancreatic tissue [20]. This suggested that miRNA up-regulation represents an important event for pancreatic cancer progression, but interestingly comparing the miRNA expression levels between the low malignant potential BCT and PDAC, general miRNA down-regulation in cancer was observed (Table S3).

**Figure 1:**
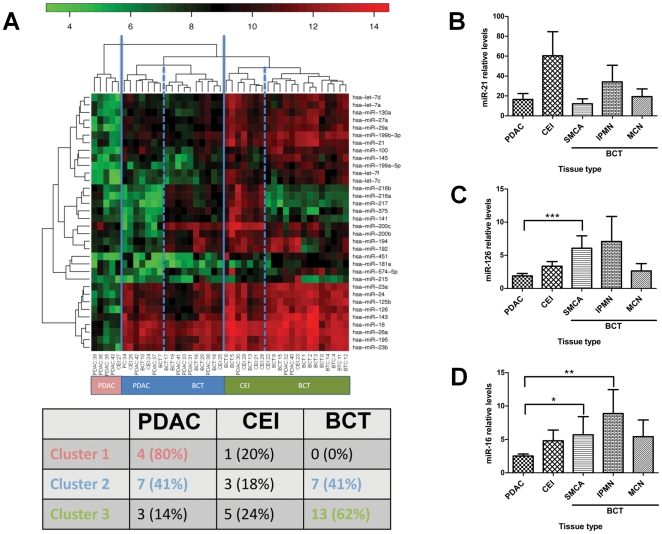
A subset of miRNAs are down-regulated in PDAC compared to Benign Cystic Tumors (BCT). (A) Hierarchical Clustering Heatmap was created to detect possible clusters in rows (transcripts) and columns (samples) of the normalized expression matrix. For this analysis we used the 35 miRNAs with highest overall variability. As the heatmap, with its dendrogram on top and the contingency table at the bottom, shows we detect three clusters indicated by the solid blue lines. The first cluster consists of 80% PDAC, 20% CEI and no BCT samples and thus contains predominantly PDAC samples. The second cluster contains 41% PDAC, 41% BCT and 18% CEI samples. Subdividing it into two additional clusters, as indicated by the dashed blue lines, we see that the left part consists predominantly of CEI, while the right part entails a slight enrichment for BCT samples. Finally, the third cluster contains 14% PDAC, 24% CEI and 62% BCT samples, thus consists predominantly of BCT samples (*P* = 0.034). (Red indicates high intensity; green indicates low intensity; PDAC, Pancreatic Ductal Adenocarcinoma; CEI, Carcinoma-Ex-IPMN; BCT, Benign-Cystic-Tumors). (B) *miR-21* (C) *miR-126* and (D) *miR-16* were measured using RT-qPCR, performed on the 43 FFPE tissues in order to validate the microarray data. Samples included: SMCA (n = 7), MCN (n = 6), IPMN (n = 7), and CEI (n = 9) and PDAC (n = 14). (Results presented as mean±SEM; *** *P* = 0.003, ** *P* = 0.02 and * *P* = 0.05 respectively).

Hierarchical clustering based on the expression of these miRNAs correctly aggregated benign and PDAC cases. The first cluster consists of 80% PDAC, 20% CEI and no BCT samples and thus contains predominantly PDAC samples. The second cluster contains 41% PDAC, 41% BCT and 18% CEI samples and finally the third cluster contains 14% PDAC, 24% CEI and 62% BCT samples, thus consists predominantly of BCT samples (Figure 1A). The detected partitioning and clustering was statistically significant (*P* = 0.034).

Next, miRNA expression profiles of PDAC were compared with different types of BCT (Tables S3, S4, S5, S6) to observe whether it would be possible to distinguish between them. Although no significant differential expression of miRNAs was identified between the BCT subgroups (i.e. IPMN vs. MCN or SMCA; IPMN vs. CEI), 21 miRNAs were down-regulated and none were up-regulated in PDAC compared to SMCA (low malignant potential BCT).

### RT-qPCR validates the microarray results

To confirm the microarray results, Taqman RT-qPCR and normalized miRNA expression levels by snRNA U6, snoRNA U47 and also by *miR-191* (as it did not change across tumor type in the microarray) were used. All of the controls reached the same statistical significance. Since their deregulation is important for cancer progression, *miR-21* [35]–[36], *miR-126* [37] and *miR-16* [38] were selected for further analysis using RT-qPCR, furthermore *miR-126* and *miR-16* have not been well studied in PDAC. RT-qPCR was performed with the same RNA as in the microarray. This revealed that although as expected there was no significant change of *miR-21* between the BCT types (Figure 1B), *miR-126* and *miR-16* were significantly down-regulated in PDAC compared to SMCA (low malignant potential BCT) (Figures 1C and D).

### MiR-21 is up-regulated in PDAC and SMCA compared to non-tumor samples

As *miR-21* is well described as being up-regulated in PDAC compared to normal tissues [20], we used normal pancreas to confirm the up-regulation of *miR-21* in PDAC and to examine expression levels of the other selected miRNAs.

**Figure 2:**
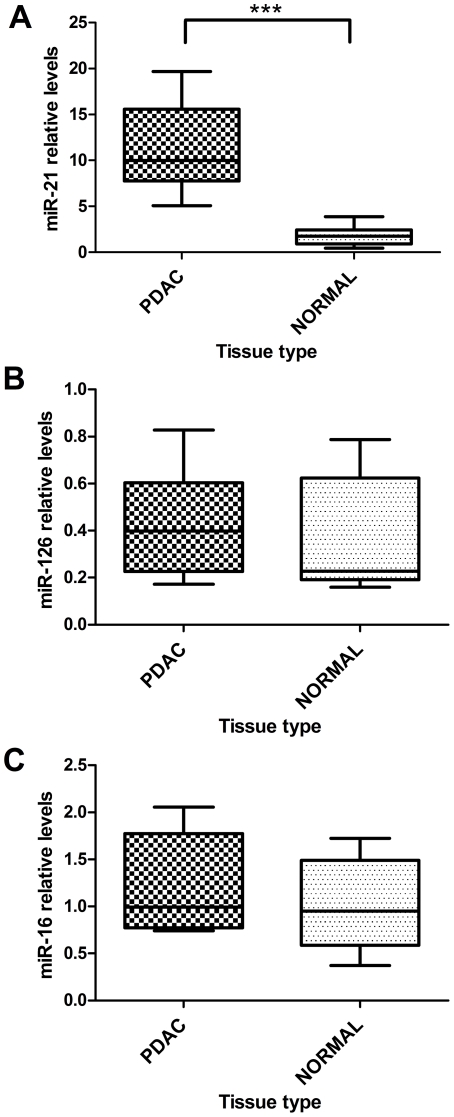
RT-qPCR of selected miRNAs performed on fresh tissues. (A) *miR-21*, (B) *miR-126* and (C) *miR-16* were measured using RT-qPCR in order to compare PDAC to normal pancreas for the miRNAs of interest. *MiR-21* is overexpressed in PDAC compared to normal pancreas tissue (*** *P*<0.001). *MiR-126* and *miR-16* expression levels were not significantly different between PDAC and normal pancreas tissue. Samples included: normal pancreas (n = 9) and PDAC (n = 15). Box and Whiskers indicate median, minimum and maximum.

RNA from a panel of fresh non-tumorous and PDAC tissues samples (n = 24) was extracted in order to measure miRNA expression levels by RT-qPCR. We confirmed that *miR-21* was significantly up-regulated in PDAC (*P*<0.001) compared to normal pancreas (Figures 2A). Furthermore, no significant changes were found in the expression levels of *miR-126* and *miR-16* between fresh normal pancreas and PDAC tissue (Figures 2B and C), but as confirmed by RT-qPCR, there was significant down-regulation of *miR-126* and *miR-16* between SMCA (low malignant potential BCT) and PDAC in the FFPE samples (Figures 1C and D).

**Figure 3:**
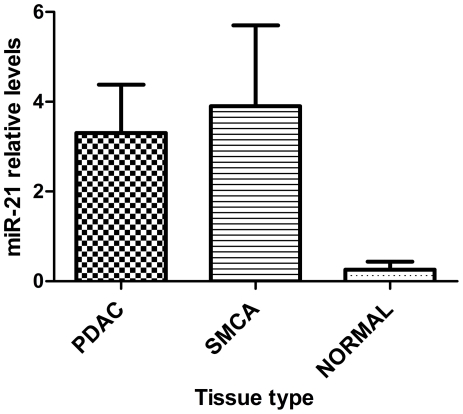
RT-qPCR confirmed *miR-21* overexpression in PDAC and SMCA compared to normal pancreas. This suggests that *miR-21* overexpression may be an early event in the formation of pancreatic BCT from normal pancreas. *MiR-21* was unable to differentiate PDAC from SMCA and therefore it may be questionable as a future biomarker of PDAC. RNA was isolated from FFPE samples for all 3 tissue types. (Results presented as mean±SEM).

In order to make a comparison with the FFPE BCT, we paraffinized some of the normal fresh pancreas samples for RNA extraction and RT-qPCR validation. Interestingly, in these FFPE samples we confirmed that *miR-21* was up-regulated in PDAC (n = 14), as well as in SMCA (n = 7), compared to normal pancreas (n = 4) (Figure 3). This indicates that the expression of *miR-21* is an early event able to increases pancreatic cell proliferation, but not malignant transformation.

### MiR-16, miR-126 and let-7d modulate the expression of pancreatic cancer oncogenes

The current study has revealed that many of the miRNAs found to be down-regulated in PDAC compared to SMCA (low malignant potential BCT) can potentially regulate the expression of genes which promote malignant transformation. PDAC is characterized by the deregulation of many proto-oncogenes among which KRAS, HMGA2, BCL2 and CRK are critical [39], [40], [41], [42]. The great majority of PDAC cases harbor a gain-of-function mutation of KRAS that results in the generation of a constitutively active form [39]. We show that KRAS and BCL2 protein are significantly up-regulated in PDAC patients (Figure S2A and B). However no significant change was observed at the mRNA level for KRAS (Figure S2C), suggesting a post-transcriptional regulation in PDAC that could be mediated by the miRNA pathway. Moreover, it is already known that KRAS and HMGA2 are regulated by the *let-7* family (family of miRNAs that we found to be down-regulated in PDAC in our microarray (Table S3)) in PDAC and other cancers [43]. The levels of KRAS mRNA have also been found to vary randomly in colorectal cancer despite consistent up-regulation of KRAS protein expression [44]. It is also known that *miR-16* regulates BCL2 expression and acts as a tumor suppressor in prostate cancer and chronic lymphocytic leukemia (CLL) [45], [46]. Furthermore, studies have shown that administration of the precursor to *miR-16* into a murine model of metastatic prostate cancer results in attenuation of disease progression [47]. Interestingly, it has been shown that *miR-126* directly regulates the expression of CRK in non-small cell lung carcinoma [48], gastric [49] and breast cancer [50] and one would expect PDAC to exhibit high expression of CRK if this oncogene is repressed by *miR-126* in pancreas. However, we could not find any significant difference in CRK protein levels when comparing normal pancreas and PDAC patient samples (Figure S2A and B). CRK is a component of the focal adhesion complex that is involved in integrin signalling and high levels of CRK have been associated with an aggressive phenotype of carcinomas [50]. We therefore performed immunohistochemical analysis using a larger sample size, also containing SMCA cases, to stain for CRK protein directly on the tissue. This identified increased CRK protein levels in PDAC compared to benign tissues and normal, indicating a regulatory role of *miR-126* in this tumor type. Representative sections of CRK protein levels in the different pancreatic tissues can be seen in Figure S3. Analysis using Fisher's Exact test indicated a statistically significant difference in CRK staining intensity between PDAC, normal pancreas and SMCA (*P* = 0.0048).

**Figure 4:**
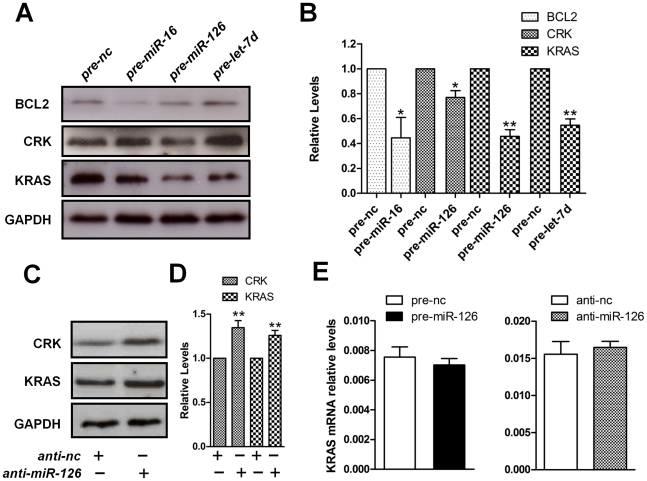
Down-regulated miRNAs allow increased expression of crucial PDAC oncogenes. (A) Western Blots showing expression levels of BCL2, CRK and KRAS oncogenes in MIA PaCa-2 cells. Transfection was performed for 48 hours with precursor *miR-16*, *miR-126* and *let-7d*. GAPDH was used as an endogenous loading control. These are representative blots derived from three biological replicates (nc, negative control). (B) Densitometric western analysis. Bar diagram of density ratio (protein/GAPDH). Negative control (nc) was set to 1 (*P<0.05; **P<0.01). (C) Western Blots showing increased expression of CRK and KRAS oncogenes in MIA PaCa-2 cells after silencing of *miR-126* using anti-miR (100 nM) transfection for 48 hours; (D) Densitometric Western analysis. Bar diagram of density ratio (protein/GAPDH). Negative control (nc) was set to 1 (*P<0.05; **P<0.01). (E) The relative expression of KRAS mRNA after pre-miR-126 or anti-miR-126 was analyzed using RT-qPCR. GAPDH was used as a housekeeping control. All data are shown as mean±SD.

In order to evaluate whether any of these miRNAs down-regulate the expression of these oncogenes in PDAC, the miRNAs were first over-expressed, by transfecting mimics into MIA PaCa-2 and PANC-1 PDAC cell-lines followed by Western blot analysis. Over-expression of pre-miR-16 down-regulates BCL2 expression compared to the over-expressed negative control (Figure 4A, 4B and Figure S4A). Furthermore CRK levels were reduced by pre-miR-126 transfection (Figure 4A, 4B and Figure S4A) and surprisingly, KRAS was down-regulated not only by pre-let-7d, but also by pre-miR-126 in MIA PaCa-2 cells (Figure 4A and 4B). As it is well documented that the tumour-suppressor *let-7* family regulates KRAS in pancreatic [51], [52], lung [53], colon [54] and breast cancers [55], we concentrated on *miR-126* as a novel KRAS targeting miRNA in PDAC. To this end we performed loss of function experiments using specific miRNA inhibitors to further validate this finding. We could demonstrate that in contrast to pre-miR-126 expression, the down-regulation of *miR-126* increases both KRAS and CRK protein levels (Figure 4C and 4D). Since we could not see any difference in KRAS mRNA levels using either pre-miR-126 or anti-miR-126, this indicates that this miRNA possibly acts on the protein translation step (Figure 4E). The data herein demonstrate that the down-regulation of multiple miRNAs in PDAC may contribute to malignant transformation.

### MiR-126 regulates KRAS protein translation by interacting with a “seedless” motif in its 3′UTR

**Figure 5:**
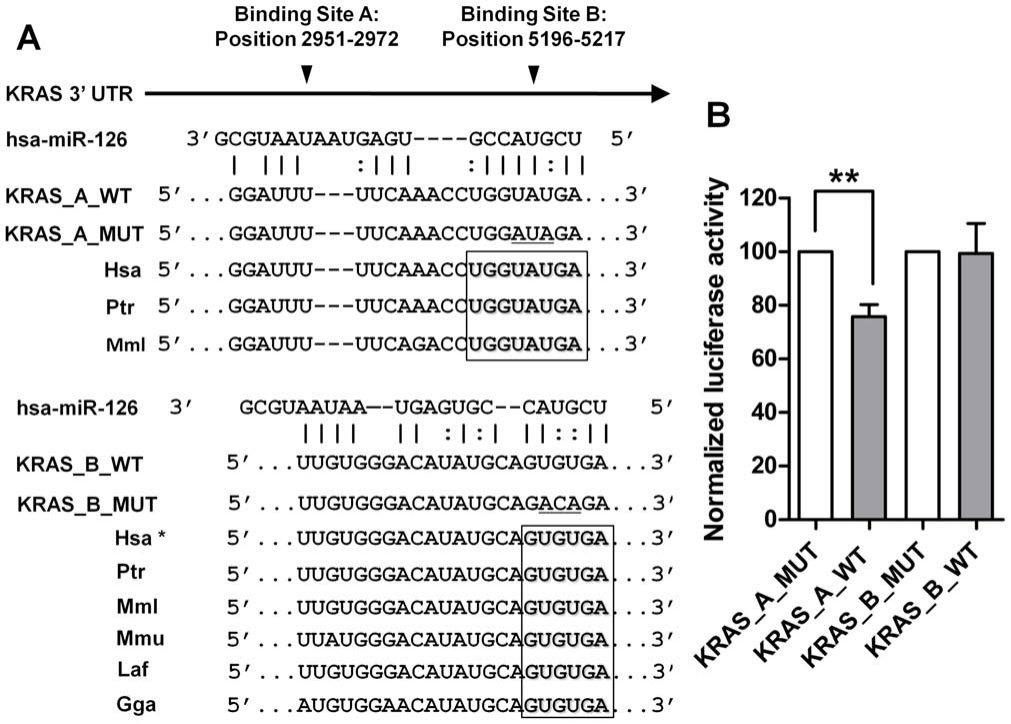
KRAS is experimentally validated as a direct target of *miR-126* in pancreatic cancer cells. (A) Putative *miR-126* binding sequences in the 3′-UTR of KRAS mRNA. Two different fragments from the 3′-UTR region of KRAS were cloned downstream of the luciferase reporters and named as wild-type (KRAS_A_WT and KRAS_B_WT). Two mutated versions of the *miR-126* binding site were also generated (KRAS_A_MUT and KRAS_B_MUT); the mutated nucleotides of the *miR-126* binding site are underlined. Boxed areas represent conserved complementary nucleotides of the *miR-126* seed sequence in various species (Hsa, human; Ptr, chimpanzee; Mml, rhesus; Mmu, mouse; Laf, elephant; Gga, chicken). *indicates that KRAS_B_WT is conserved in 16 species. (B) Luciferase reporter assay. Each of the 4 plasmids (150 ng) and a *Renilla* luciferase reporter (50 ng) were co-transfected into MIA PaCa-2 cells with precursor miR-126 (100 nM). Luciferase activity was assayed 48 hours after transfection. All experiments were independently repeated at least three times; the results are presented as mean±SD (**P<0.01).

We show that over-expression of *miR-126* reduces and conversely its silencing increases KRAS protein levels (Figure 4A, 4B, 4C and 4D). In order to evaluate whether *miR-126* directly regulates KRAS, we performed a bioinformatic search of potential *miR-126* interaction sites in the KRAS mRNA. Using the RNA22 software [56] and the entire KRAS transcript as the input sequence, we predicted two *miR-126* binding sites in the 3′UTR with “seedless” characteristics (Figure 5A). This means that these interaction sites do not have canonical features of complete interaction between the 5′ seed region of the miRNA [57] and the 3′UTR of the gene that has been indicated to be important for the regulation of the target genes [58]. But instead G-U wobbles were present in the complementarity between gene and seed miRNA sequence (Figure 5A). Interestingly, these two regions appeared evolutionally conserved across species (Figure 5A) and more importantly it has been recently demonstrated that miRNAs can regulate gene expression also using “seedless” pairing [58]. For these reasons we went on to clone the two sites that we termed KRAS_A_WT and KRAS_B_WT into the 3′UTR of pMIR-REPORT construct along with a mutated version of each (Table S2) and co-expressed them with the pre-miR-126 in MIA PaCa-2 cells. Over-expression of *miR-126* decreased luciferase activity only when co-expressed with KRAS_A_WT and not the mutated version (KRAS_A_MUT) (Figure 5B). This indicates that *miR-126* directly regulates KRAS at post-transcriptional levels through a “seedless” interaction with its 3′UTR.

## Discussion

Although the pancreas specific miRNAome and how it is modified in PDAC has been extensively investigated [59], only a limited number of studies have looked at miRNA expression in pancreatic pre-malignant lesions [60], [61] indicating an urgent need for further investigation. Du Rieu *et al*. examined samples of non-pathologic pancreatic ducts and microscopic pancreatic intraepithelial neoplasia (PanIN) precursor lesions from a KRAS (G12D) mouse model and from human FFPE samples adjacent to PDAC. They showed that *miR-21* deregulation occurs in the most advanced PanIN-3 lesions, before they become invasive PDAC [60]. Habbe et al. looked at the expression of 12 selected miRNAs in IPMN compared to normal pancreas and CEI [61]. They found 10 miRNAs significantly up-regulated in IPMN compared to normal pancreas; of which *miR-21* and *miR-155* were identified as possible biomarker candidates for PDAC progression from normal pancreas to IPMN to adenocarcinoma.

For the first time, we have examined global miRNA expression in all the epithelial macroscopic pre-malignant pancreatic BCT (i.e. SMCA, MCN and IPMN), compared to PDAC and CEI, by microarray to reveal the miRNA-based relationship between these lesions. Interestingly, with a few exceptions, PDACs tend to cluster together and remain well separated from the BCT.

There were no significant changes in the miRNA expression patterns between the various types of BCT, indicating that miRNA expression changes were not involved in transitions between the BCT types and more importantly that such transitions were unlikely to occur *in vivo*. A widespread miRNA down-regulation in PDAC was observed compared to SMCA, the most benign lesion that rarely progress to invasive adenocarcinoma. We observed that many of the miRNAs down-regulated in PDAC belong to the same family or cluster. Being that the probes used in the microarray are randomly located in the platform, we regard this as validation of our findings. For example, among the miRNAs that we found to be down-regulated, m*iR-15a* forms a cluster with *miR-16, miR-24* forms a cluster with *miR-23a* or *miR-27b*, *miR-29a* with *miR-29b, miR-143 with miR145* and each cluster is expressed as a unique primary transcript (Table S3).

It has widely been described that miRNA up-regulation characterizes PDAC [19], [20], whilst cancers are usually characterized by general miRNA down-regulation [62]. We confirm that *miR-21* up-regulation is actually an early event that induces normal non-proliferative cells into benign proliferative cells. Dysregulation of proteins involved in miRNA biogenesis in PDAC, which still need to be characterized, could explain this event. Among the down-regulated miRNAs in our microarray, there are many already described as tumor suppressors through inhibition of known PDAC oncogenes. We show general *miR-29* family member down-regulation. Amongst their targets are DNMT3A and 3B-methyltransferases, whose levels can increase because of the loss of *miR-29*, causing CpG island hypermethylation and cancer [63]. We also show down-regulation of *let-7* family members (*let-7f, let-7d, let-7c, let-7a, let-7i*) (Table S3), which are already described as negative regulators of KRAS and HMGA2 oncogenes, whose increased activity is very important during PDAC progression [53], [64]. Furthermore, we show down-regulation of both *miR-143* and *miR-145*, which have recently been described as being transcriptionally down-regulated by the Ras signaling pathway, that in turn directly targets KRAS oncogene in PDAC [28]. This revealed a feed-forward mechanism that potentiates Ras signaling [28]. This was of interest as it is well known that KRAS is one of the main genetic promoters of PDAC [39] and HMGA2 expression levels are associated with the malignant phenotype in pancreatic exocrine tissue [42], which could in part be explained by the down-regulation of these miRNAs. Interestingly, we could see an up-regulation of KRAS protein, but no change in mRNA levels when comparing normal tissues to PDAC, indicating that the post-transcriptional regulation of KRAS in PDAC may be an essential step.

Mutations that result in a constitutively active KRAS are found in >95% of PDAC and are thought to be a crucial initiating event for this disease [65]. Furthermore, PDAC continues to be “addicted” to KRAS for epithelial differentiation and cell viability, indicating that finding new KRAS regulators is an important step [66]. We show a down-regulation of *miR-126* in PDAC, with increased expression of KRAS. As a result, we evaluated a possible role for *miR-126* in regulating KRAS and found that it is able to directly regulate KRAS, inhibiting its protein translation by interacting with a “seedless” site within its 3′UTR. This suggests that its downregulation in PDAC could participate in the progression of PDAC because of the subsequent KRAS increase. *MiR-126* expression was in fact down-regulated in PDAC compare to SMCA (a low malignant potential BCT) and previous studies have shown that these BCT lesions are devoid of the KRAS mutation [67], [68]. The high malignant potential BCT (i.e. IPMN and MCN) have been shown to have the mutated KRAS more frequently [69], [70] and we show these lesions had no significant difference in *miR-126* expression when compared to PDAC. Interestingly, for progression from PanIN to BCT to adenocarcinoma these mucinous lesions require KRAS (G12D), followed by loss of heterozygosity of SMAD4 and mutation of p53 or p16 [71]. As we show *miR-126* up-regulation occurs in SMCA, this raises the possibility of replacement miRNA therapy for those patients with low *miR-126* in their BCT at the time of pre-operative biopsy or even as adjuvant treatment after surgical resection to prevent recurrence or control disease.

*MiR-16* is often down-regulated in chronic lymphocytic leukaemia [72], gastric [73], ovarian [74] and prostate cancers as a tumor suppressor that targets and down-regulates the anti-apoptotic gene BCL2 [45]. *MiR-126* is down-regulated in various tumors compared to non-cancerous tissues including breast, lung, stomach, cervix, bladder, and prostate [37]. Recently, *miR-126* has been shown to be a tumor suppressor in gastric cancer as it can inhibit tumor growth and metastasis *in vivo* and *in vitro*. This effect was partially mediated by down-regulation of CRK [49]. SRC and CRK-associated substrate phosphorylation is an important promoter of PDAC anchorage-independence and tumor progression [41]. SRC is able to repress *miR-126* expression levels [50] and furthermore *miR-126* has been described as a suppressor of proliferation and metastasis in breast cancer [75]. We have established that *miR-16* targets BCL2 and *miR-126* targets at least CRK and KRAS in PDAC cell-lines. As already shown, we did not observe any significant change in *miR-16* and *miR-*126 expression comparing normal pancreas to PDAC using RT-qPCR, but did find significant down-regulation of both miRNAs in PDAC compared to a low malignant potential BCT. Whilst the down-regulation of *miR-16* has not been seen previously in PDAC compared to normal pancreas [76], the reduction of *miR-126* in PDAC has recently been reported [77]. As both are frequently down-regulated in several tumor types, their importance in tumorigenesis is clear.

We could not see *miR-21* as up-regulated in PDAC compared to SMCA. Croce's group have also examined the *oncomiR-21* in more detail in 80 PDAC specimens and found that it is significantly overexpressed in PDAC, but that its expression does not correlate with tumor size, nodal status or T stage [1]. We observed that its up-regulation from normal tissue is almost certainly a very early event that occurs in the low malignant potential BCT we studied and this occurs even earlier than previously described [60], [61]. This suggests that *miR-21* induces pancreatic cell proliferation, but it is not sufficient to induce malignant transformation. Since *miR-21* has recently been demonstrated to be up-regulated in PDAC compared to normal tissue [20] and we show here that it is not deregulated in PDAC compared to pre-malignant BCT, this indicates that its up-regulation is likely to be an early event important for benign neoplasm formation from normal tissue.

**Figure 6:**
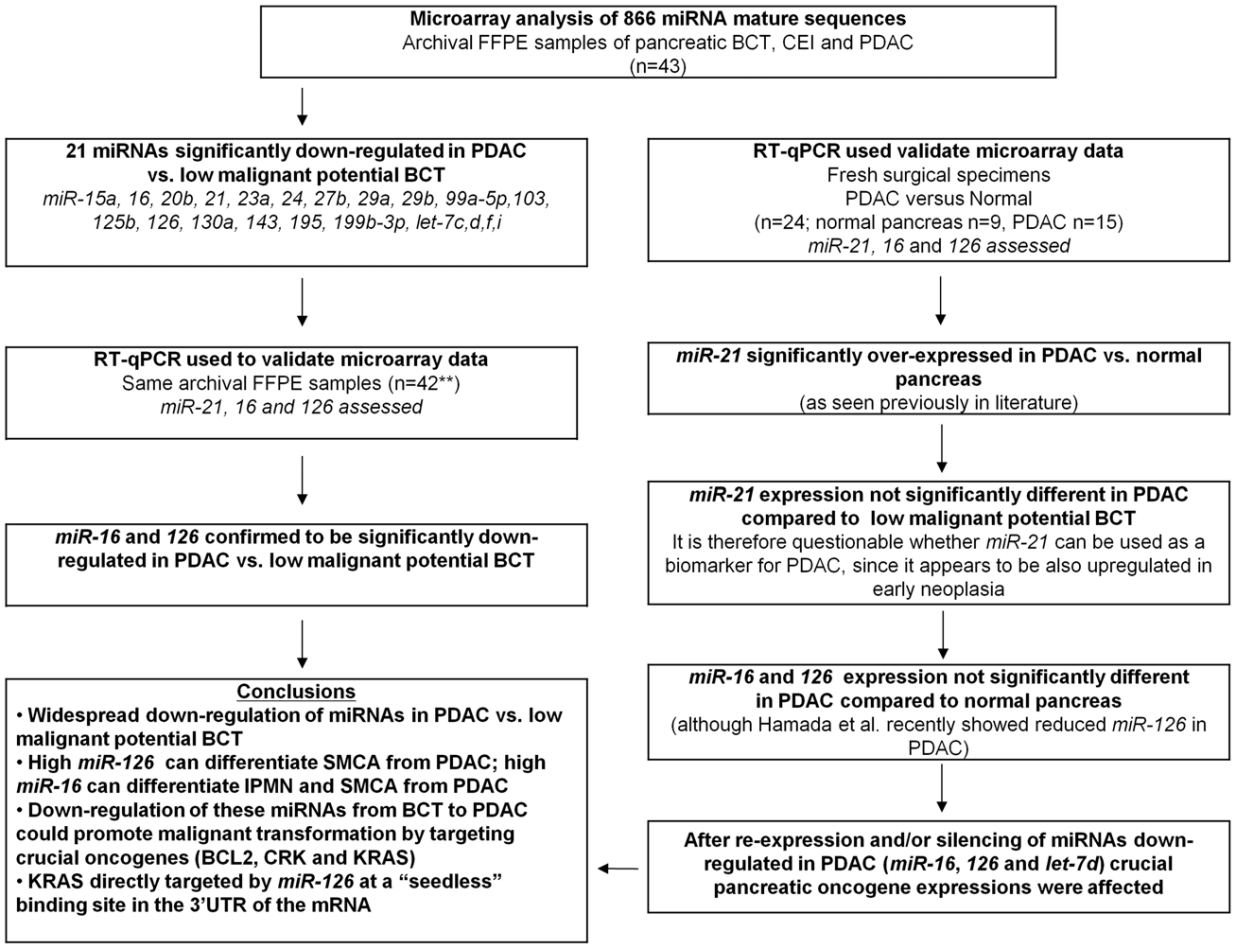
Flow chart of miRNA expression profiling in pancreatic BCTs and miRNA target acquisition. (FFPE, formalin-fixed paraffin embedded tissue; **indicates that RNA could not be isolated from 1 sample; RT- qPCR, quantitative reverse transcription polymerase chain reaction).

The differential diagnosis of pancreatic BCT remains a clinical challenge. A better understanding of the natural history of these lesions is considered central to understanding the risk of malignant transformation. We observed significantly down-regulated miRNAs in PDAC compared to low malignant potential BCT, such as *miR-16*, *miR-126* and *let-7d*, which could be confirmed by qRT-PCR and target known PDAC oncogenes such as BCL2, CRK and KRAS. We thus demonstrate that miRNAs have the potential to be used to differentiate pancreatic BCT from malignant PDAC (Figure 6). For the first time we have shown that KRAS is directly targeted by *miR-126* by binding to a “seedless” site in its 3′UTR. As the majority of PDAC are driven by activated KRAS, the re-expression of this miRNA, along with other miRNAs known to also negatively regulate this crucial oncogene (i.e. *let-7 family, miR-96* [78] and *miR-217* [79]), may provide a therapeutic strategy for treating this devastating disease.

### Limitations

Whilst there are some striking findings from the microarray and validation, the following should be taken into account. Firstly, the various pancreatic BCT are very rare (prevalence reported in the literature as between 0.2–2.6% in the asymptomatic general population [9]) and the tissues are difficult to obtain as few patients undergo surgical resection. This is reflected in our small sample sizes. Thus whilst we conclude that there are no statistically significant deregulated miRNAs between many of the groups, this may in fact be a Type II error. Secondly, this is also true of our validation of *miR-16* and *miR-126* in PDAC compared to normal pancreas. Whilst we did not see significant down-regulation for either of these miRNAs, this may also be a Type II error. Hamada et al. have recently shown that *miR-126* is down-regulated in PDAC and has tumor suppressive effects by targeting ADAM9, which enhances cancer cell invasion by modulating tumor-stromal cell interactions. Re-expression of *miR-126* reduced cellular migration and invasion in PDAC cell lines [77]. It would be appropriate to undertake further miRNA studies on the high malignant potential pancreatic lesions and validate candidate miRNAs in a larger cohort, ideally in the prospective and multicentric setting.

## Supporting Information

Figure S1 **Epithelial benign cystic tumors of the pancreas.** Our study concentrated on the tumors of epithelial origin in order to identify miRNAs which may be involved in the development of early neoplasia and pancreatic ductal adenocarcinoma (PDAC).
(TIF)

Figure S2 **BCL2, CRK and KRAS expression levels in PDAC and normal pancreatic tissue.** (**A**) Western Blots showing protein levels of BCL2, CRK, KRAS and GAPDH in PDAC (n = 9) fresh tissue samples compared to normal pancreas (n = 9). (**B**) Bar chart showing mean relative protein levels of the Western Blots analyzed by densitometric scanning after normalization to GAPDH (*BCL2 levels in PDAC vs. Normal *P* = 0.03; **KRAS levels in PDAC vs. Normal *P* = 0.0003). (**C**) RT-qPCR performed on the same fresh tissue samples showing KRAS mRNA levels in PDAC (n = 6) compared to normal (n = 6) after normalization to GAPDH.
(TIF)

Figure S3 **Immunohistochemical analysis of CRK expression in pancreatic tissues.** Paraffin sections were analyzed using anti-CRK antibody and counterstained with hematoxylin. Cytoplasmic staining (brown) was observed in PDAC and normal pancreas, but not in SMCA. Original photographs were taken at magnification 20×. Staining intensity was measured as 0 for no expression, 1+ for weak expression and 2+ for moderate expression. Bar charts indicate the % in each category for each tissue type. A 3×3 contingency table was created and analyzed using the Fisher's Exact test to reveal a significant difference between the 3 tissue types (i.e. increased CRK expression in PDAC>normal pancreas>SMCA; *P* = 0.0048).
(TIF)

Figure S4 **Expression levels of BCL2, CRK and KRAS oncogenes in PANC-1 cells.** (A) Western Blots showing protein levels of BCL2, CRK, KRAS after transfection for 48 hours with precursor *miR-16*, *miR-126* and *let-7d* (miRNA mimics). (B) The relative expression of KRAS mRNA after pre-miR-126 or anti-miR-126 transfection was analyzed using RT-q PCR and remained unchanged compared to negative control. GAPDH was used as a housekeeping control. All data are shown as mean±SD. (C) Western Blots showing protein levels of CRK and KRAS after transfection for 48 hours with miRNA inhibitor (anti-miR-126). GAPDH was used as an endogenous loading control for all blots. These are representative blots derived from three biological replicates (nc, negative control).
(TIF)

Table S1 **Clinicopathological characteristics of the patients for each tissue type.** MiRNA expression profiling and validation was performed on 58 pancreatic tumor samples; 43 formalin-fixed paraffin-embedded (FFPE) tumour samples were analyzed by miRNA microarray and RT-qPCR using Taqman probes; a further 24 fresh surgical specimens (normal pancreas n = 9 and PDAC n = 15) were used to validate the results using RT-qPCR. Samples available for immunohistochemical (IHC) analysis were normal pancreas n = 12, PDAC n = 12 and SMCA n = 12. Non-tumorous tissue was obtained during pancreatic trauma surgery. Key: SMCA, serous microcystic adenoma; MCN, mucinous cystic neoplasm; PDAC, Pancreatic Adenocarcinoma; IPMN, Intraductal papillary mucinous neoplasm; CEI, Carcinoma-ex-IPMN; IQR, interquartile range; *Non-disease related death (cardiac disease), RT-qPCR, quantitative reverse transcription polymerase chain reaction.
(DOC)

Table S2 **Sequences of all primers used for KRAS luciferase plasmid construction.** Red ends indicate sequences appropriate for the Mlu1 and HindIII restriction enzymes. Yellow highlighted areas indicate mutated nucleotides.
(DOC)

Table S3 **Microarray results for PDAC vs. Serous Microcystic Adenoma (SMCA).** The 30 most deregulated probes (detected by highest absolute value of logarithmized fold changes) for PDAC vs. SMCA (low malignant potential tumor). There is widespread down-regulation of miRNAs in PDAC (limma adjp indicates the *p*- value adjusted for multiple testing).
(DOC)

Table S4 **Microarray results for PDAC vs. Mucinous Cystic Neoplasm (MCN).** The 30 most deregulated probes (detected by highest absolute value of logarithmized fold changes) for PDAC vs. MCN (high malignant potential tumor). No significant difference in miRNA expression profile was shown between these 2 tissue types (limma adjp indicates the *p*-value adjusted for multiple testing).
(DOC)

Table S5 **Microarray results for PDAC vs. Intraductal Papillary Mucinous Neoplasm (IPMN).** The 30 most deregulated probes (detected by highest absolute value of logarithmized fold changes) for PDAC vs. IPMN (high malignant potential tumor). No significant difference in miRNA expression profile was shown between these 2 tissue types (limma adjp indicates the *p*-value adjusted for multiple testing).
(DOC)

Table S6 **Microarray results for Carcinoma Ex-IPMN (CEI) vs. PDAC.** The 30 most deregulated probes (detected by highest absolute value of logarithmized fold changes) for CEI (carcinoma on background of IPMN lesion) vs. PDAC. No significant difference in miRNA expression profile was shown between these 2 tissue types (limma adjp indicates the *p*-value adjusted for multiple testing).
(DOC)
