# Supplementary figures and images for: MicroRNAs Targeting Oncogenes Are Down-Regulated in Pancreatic Malignant Transformation from Benign Tumors
Source: PLoS One. 2012 Feb 22;7(2):e32068. doi: 10.1371/journal.pone.0032068 (PMC3284550; doi:10.1371/journal.pone.0032068)

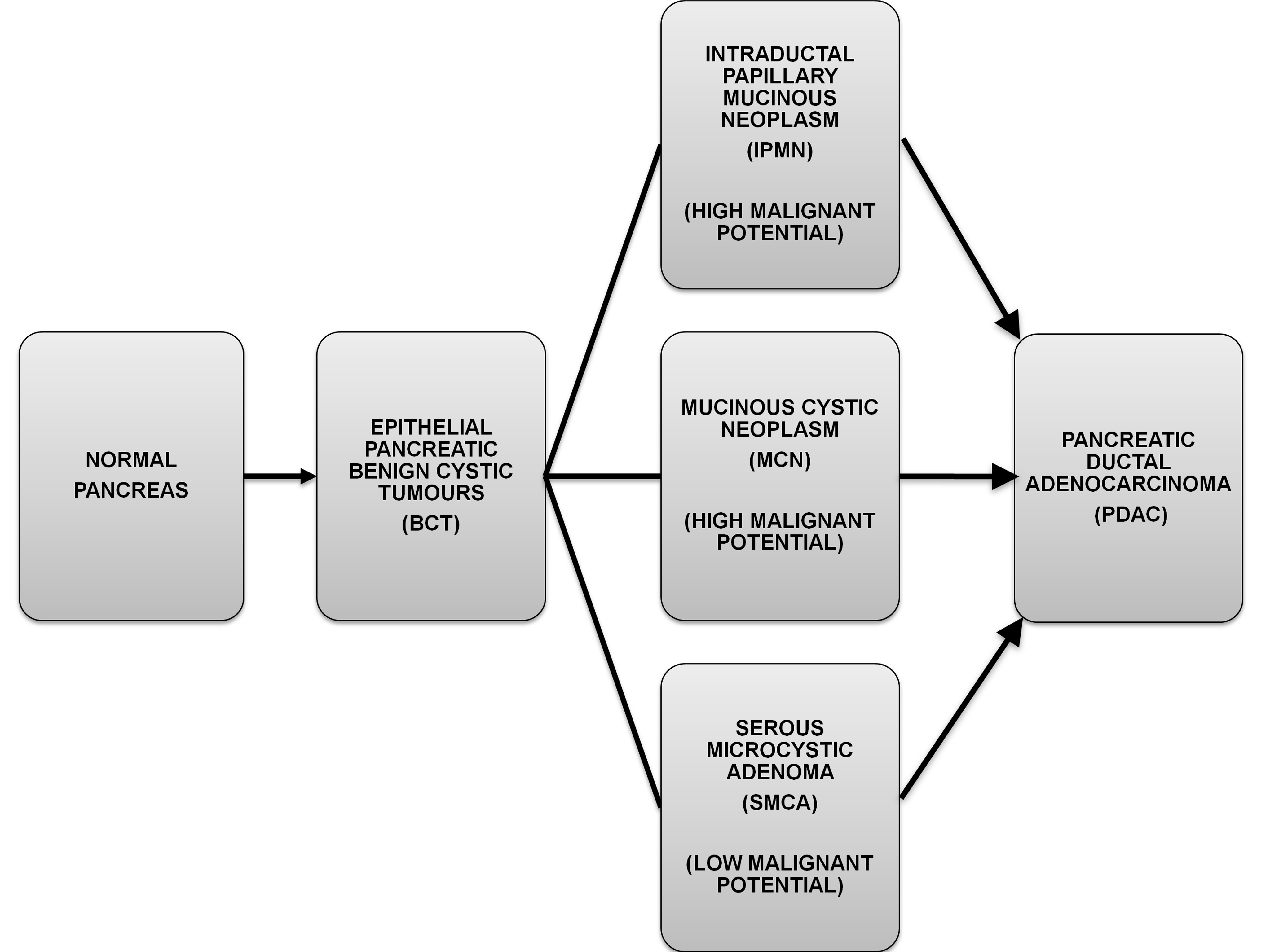

Supplement: Figure S1 — Epithelial benign cystic tumors of the pancreas. Our study concentrated on the tumors of epithelial origin in order to identify miRNAs which may be involved in the development of early neoplasia and pancreatic ductal adenocarcinoma (PDAC). (TIF) [file pone.0032068.s001.tif]

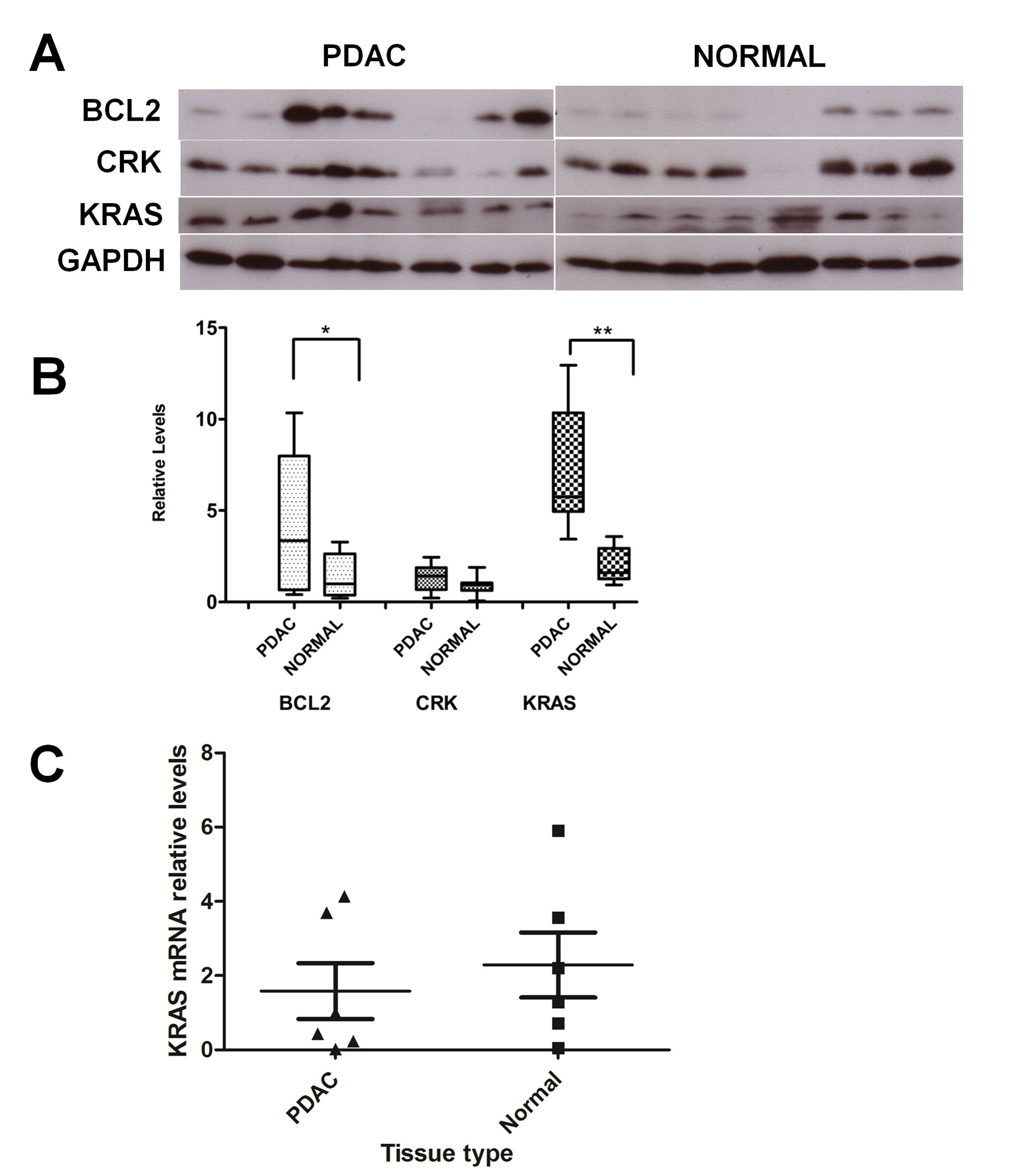

Supplement: Figure S2 — BCL2, CRK and KRAS expression levels in PDAC and normal pancreatic tissue. (A) Western Blots showing protein levels of BCL2, CRK, KRAS and GAPDH in PDAC (n = 9) fresh tissue samples compared to normal pancreas (n = 9). (B) Bar chart showing mean relative protein levels of the Western Blots analyzed by densitometric scanning after normalization to GAPDH (*BCL2 levels in PDAC vs. Normal P = 0.03; **KRAS levels in PDAC vs. Normal P = 0.0003). (C) RT-qPCR performed on the same fresh tissue samples showing KRAS mRNA levels in PDAC (n = 6) compared to normal (n = 6) after normalization to GAPDH. (TIF) [file pone.0032068.s002.tif]

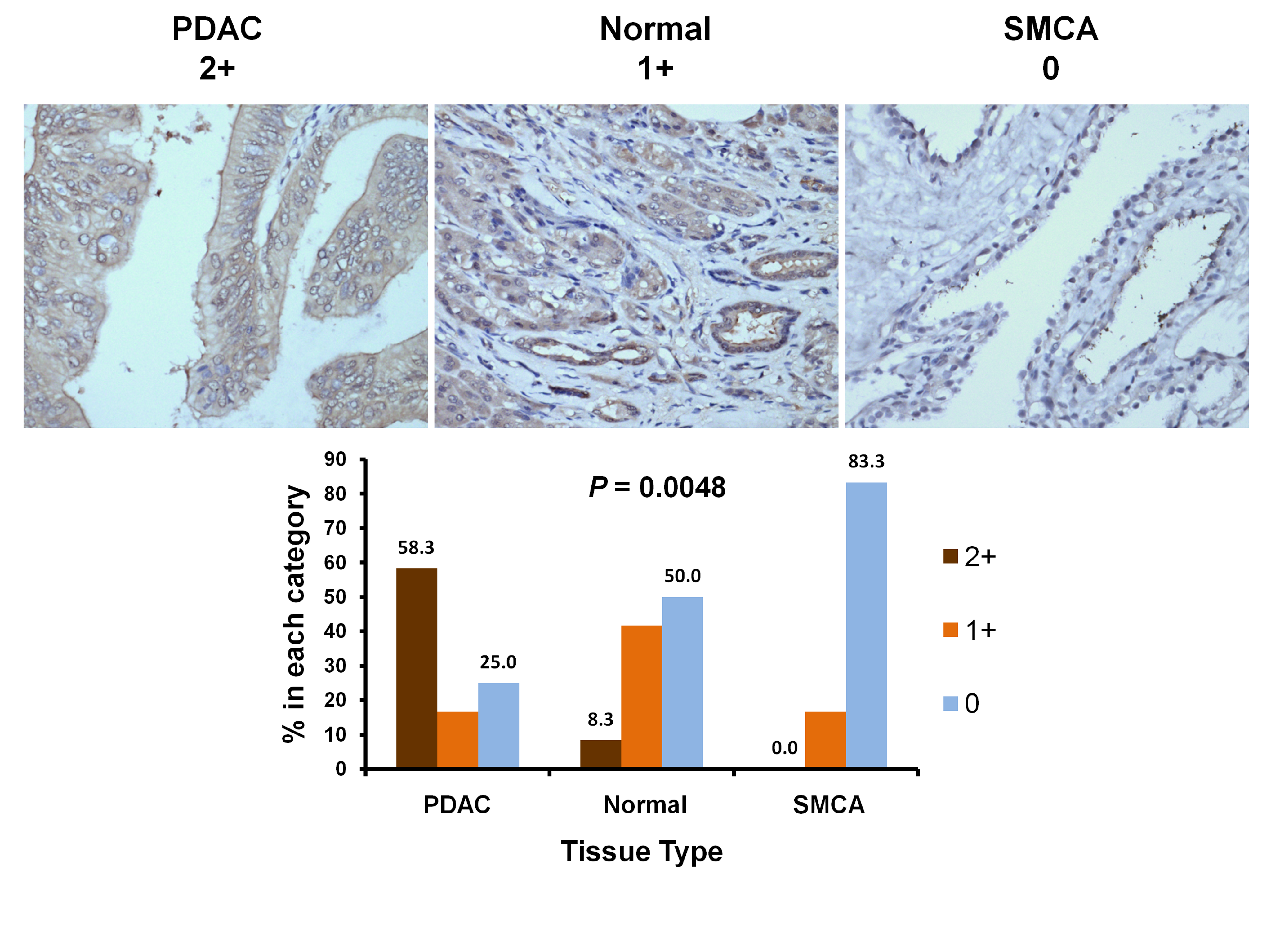

Supplement: Figure S3 — Immunohistochemical analysis of CRK expression in pancreatic tissues. Paraffin sections were analyzed using anti-CRK antibody and counterstained with hematoxylin. Cytoplasmic staining (brown) was observed in PDAC and normal pancreas, but not in SMCA. Original photographs were taken at magnification 20×. Staining intensity was measured as 0 for no expression, 1+ for weak expression and 2+ for moderate expression. Bar charts indicate the % in each category for each tissue type. A 3×3 contingency table was created and analyzed using the Fisher's Exact test to reveal a significant difference between the 3 tissue types (i.e. increased CRK expression in PDAC>normal pancreas>SMCA; P = 0.0048). (TIF) [file pone.0032068.s003.tif]

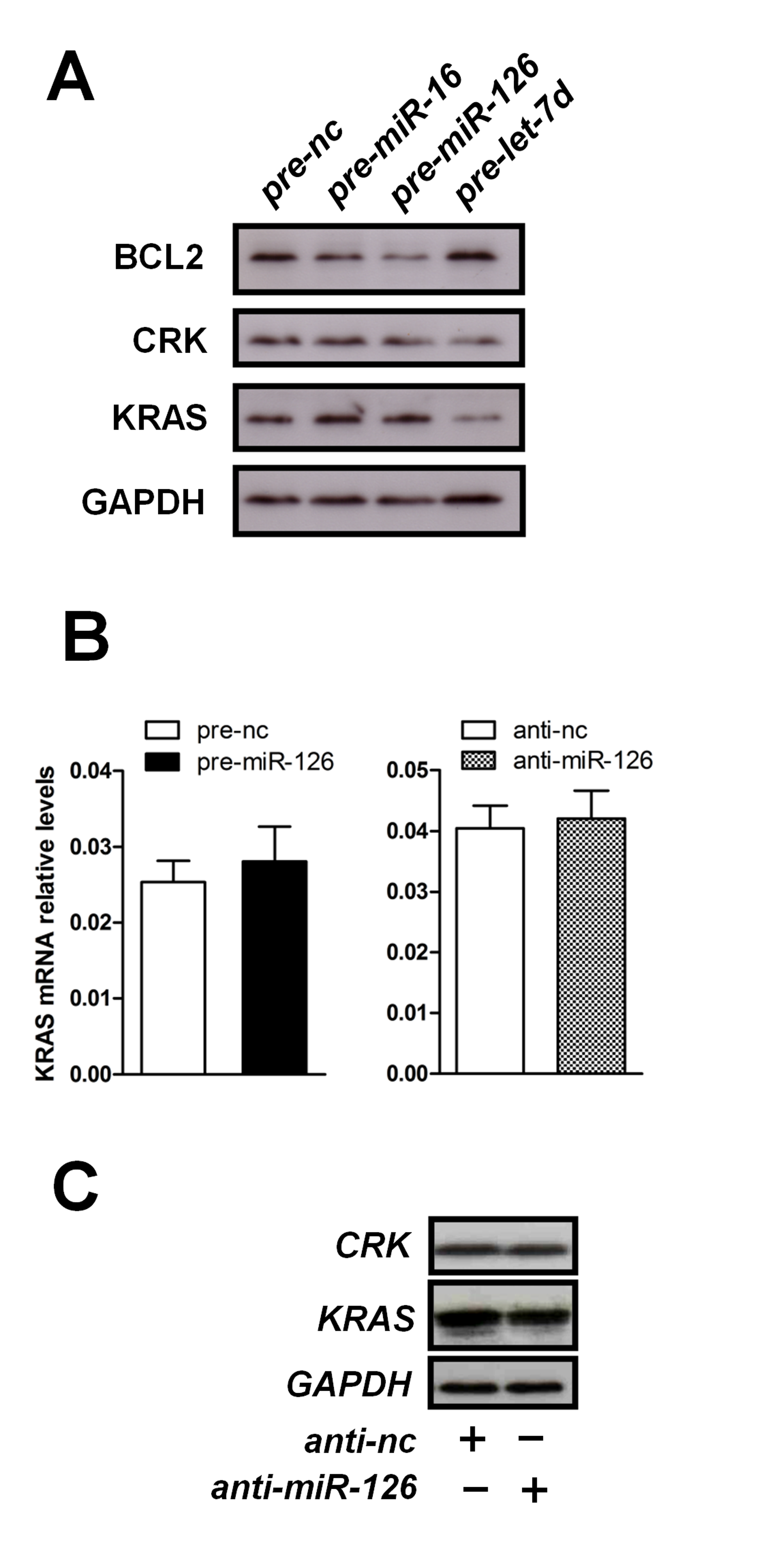

Supplement: Figure S4 — Expression levels of BCL2, CRK and KRAS oncogenes in PANC-1 cells. (A) Western Blots showing protein levels of BCL2, CRK, KRAS after transfection for 48 hours with precursor miR-16, miR-126 and let-7d (miRNA mimics). (B) The relative expression of KRAS mRNA after pre-miR-126 or anti-miR-126 transfection was analyzed using RT-q PCR and remained unchanged compared to negative control. GAPDH was used as a housekeeping control. All data are shown as mean±SD. (C) Western Blots showing protein levels of CRK and KRAS after transfection for 48 hours with miRNA inhibitor (anti-miR-126). GAPDH was used as an endogenous loading control for all blots. These are representative blots derived from three biological replicates (nc, negative control). (TIF) [file pone.0032068.s004.tif]

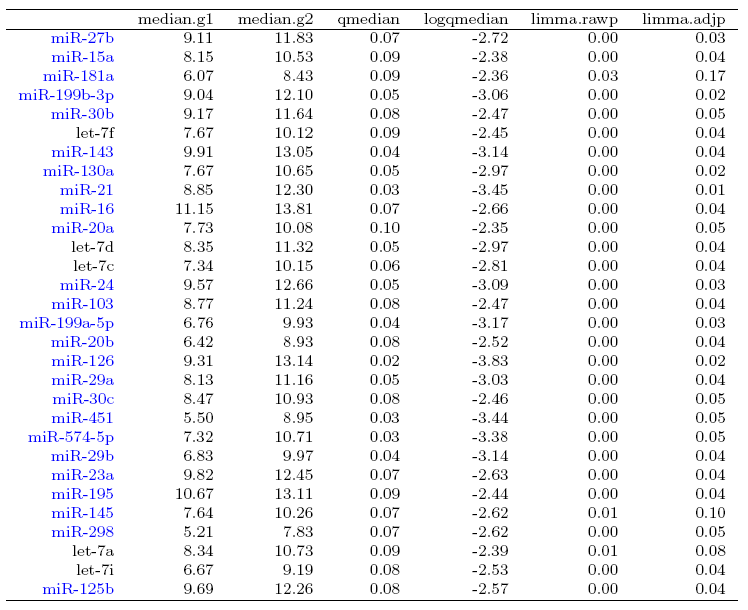

Supplement: Table S3 — Microarray results for PDAC vs. Serous Microcystic Adenoma (SMCA). The 30 most deregulated probes (detected by highest absolute value of logarithmized fold changes) for PDAC vs. SMCA (low malignant potential tumor). There is widespread down-regulation of miRNAs in PDAC (limma adjp indicates the p- value adjusted for multiple testing). (DOC) [file pone.0032068.s007.doc]

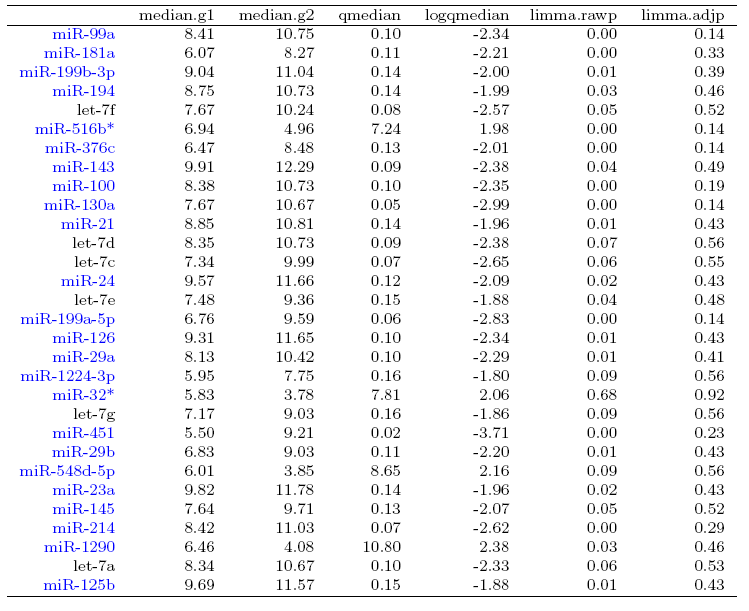

Supplement: Table S4 — Microarray results for PDAC vs. Mucinous Cystic Neoplasm (MCN). The 30 most deregulated probes (detected by highest absolute value of logarithmized fold changes) for PDAC vs. MCN (high malignant potential tumor). No significant difference in miRNA expression profile was shown between these 2 tissue types (limma adjp indicates the p-value adjusted for multiple testing). (DOC) [file pone.0032068.s008.doc]

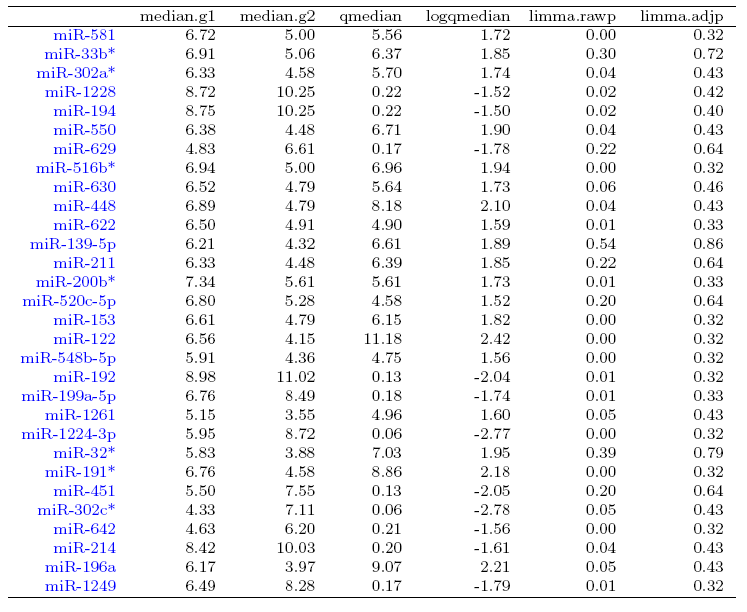

Supplement: Table S5 — Microarray results for PDAC vs. Intraductal Papillary Mucinous Neoplasm (IPMN). The 30 most deregulated probes (detected by highest absolute value of logarithmized fold changes) for PDAC vs. IPMN (high malignant potential tumor). No significant difference in miRNA expression profile was shown between these 2 tissue types (limma adjp indicates the p-value adjusted for multiple testing). (DOC) [file pone.0032068.s009.doc]

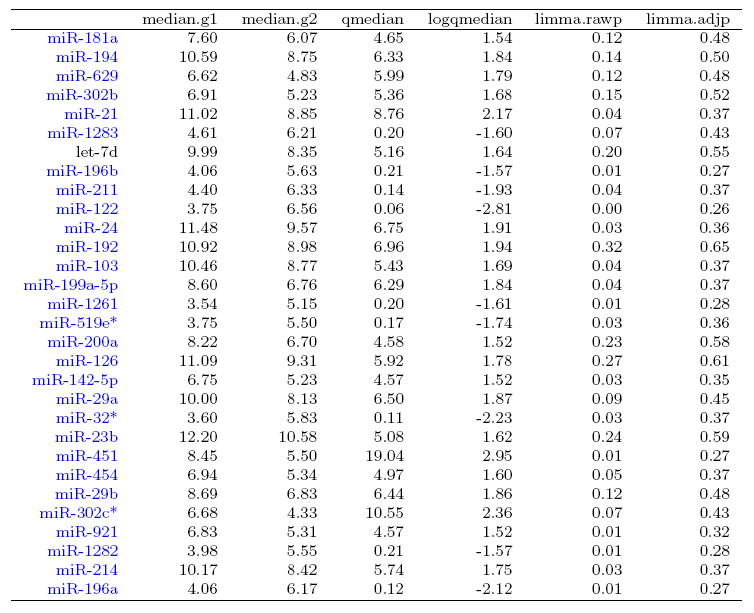

Supplement: Table S6 — Microarray results for Carcinoma Ex-IPMN (CEI) vs. PDAC. The 30 most deregulated probes (detected by highest absolute value of logarithmized fold changes) for CEI (carcinoma on background of IPMN lesion) vs. PDAC. No significant difference in miRNA expression profile was shown between these 2 tissue types (limma adjp indicates the p-value adjusted for multiple testing). (DOC) [file pone.0032068.s010.doc]
