# Supplementary material for: MicroRNAs Targeting Oncogenes Are Down-Regulated in Pancreatic Malignant Transformation from Benign Tumors
Source: PLoS One. 2012 Feb 22;7(2):e32068. doi: 10.1371/journal.pone.0032068 (PMC3284550; doi:10.1371/journal.pone.0032068)
Supplement: Table S1 — Clinicopathological characteristics of the patients for each tissue type. MiRNA expression profiling and validation was performed on 58 pancreatic tumor samples; 43 formalin-fixed paraffin-embedded (FFPE) tumour samples were analyzed by miRNA microarray and RT-qPCR using Taqman probes; a further 24 fresh surgical specimens (normal pancreas n = 9 and PDAC n = 15) were used to validate the results using RT-qPCR. Samples available for immunohistochemical (IHC) analysis were normal pancreas n = 12, PDAC n = 12 and SMCA n = 12. Non-tumorous tissue was obtained during pancreatic trauma surgery. Key: SMCA, serous microcystic adenoma; MCN, mucinous cystic neoplasm; PDAC, Pancreatic Adenocarcinoma; IPMN, Intraductal papillary mucinous neoplasm; CEI, Carcinoma-ex-IPMN; IQR, interquartile range; *Non-disease related death (cardiac disease), RT-qPCR, quantitative reverse transcription polymerase chain reaction. (DOC) [file pone.0032068.s005.doc]

|  | **BENIGN CYSTIC TUMOR** | | | | **PDAC** | | **NORMAL** | **TOTAL** |
| --- | --- | --- | --- | --- | --- | --- | --- | --- |
|  | SMCA  (FFPE n=7 in microarray; n=12 in IHC) | MCN  (FFPE n=6) | IPMN  (FFPE n=7) | | CEI  (FFPE n=9) | Adenocarcinoma  (FFPE n=14; fresh n=15) | (FFPE n=12; fresh n=9) | (PDAC n=38; Total tumors n=63) |
| **Sex** | | | | | | | | |
| Female | 8 | 6 | 4 | | 6 | 13 | 6 | 37 (58.7) |
| Male | 4 | 0 | 3 | | 3 | 16 | 7 | 26 (41.3) |
| **Age (median, range)** | 63 (39-78) | 50 (24-72) | 68 (53-77) | | 70 (41-76) | 67 (30-82) | - | - |
| **Tumour Size (cm; median, IQR)** | 4.3 (3.3) | 3.0 (8.2) | 2.4 (4.6) | | 3.3 (2.4) | 2.7 (1.5) | - | - |
| **Tumour (pT) stage (%)** | | | | | | | | |
| 0 | - | - | | - | 0 | 0 | - | 0 |
| 1 | - | - | | - | 0 | 1 (3.4) | - | 1 (2.6) |
| 2 | - | - | | - | 1 (11.1) | 1 (3.4) | - | 2 (5.3) |
| 3 | - | - | | - | 7 (77.8) | 14 (48.3) | - | 21 (55.3) |
| 4 | - | - | | - | 0 | 9 (31.0) | - | 9 (23.7) |
| Biopsy only | - | - | | - | - | 3 (10.3) | - | 3 (7.9) |
| Missing | - | - | |  | 1 (11.1) | 1 (3.4) | - | 2 (5.3) |
| **Nodal Status (%)** | | | | | | | | |
| Negative | - | - | | - | 5 (55.6) | 8 (27.5) | - | 13 (34.2) |
| Positive | - | - | | - | 4 (44.4) | 17 (58.6) | - | 21 (55.3) |
| Biopsy only | - | - | | - | - | 3 (10.3) | - | 3 (7.9) |
| Missing | - | - | | - | - | 1 (3.4) | - | 1 (2.6) |
| **Resection Status (%)** | | | | | | | | |
| R0 | - | - | | 4 (57.1) | 5 (55.6) | 15 (48.2) | - | 24 (63.2) |
| R1 | - | - | | 3 (42.9) | 4 (44.4) | 11 (37.9) | - | 18 (47.4) |
| Biopsy only | - | - | | - | - | 3 (10.3) | - | 3 (7.9) |
| **Perineural Invasion (%)** | | | | | | | | |
| Negative | - | - | | - | 3 (33.3) | 5 (17.2) | - | 8 (21.1) |
| Positive | - | - | | - | 5 (55.6) | 18 (62) | - | 23 (60.5) |
| Biopsy only | - | - | | - | - | 3 (10.3) | - | 3 (7.9) |
| Missing | - | - | | - | 1 (11.1) | 3 (10.3) | - | 4 (10.5) |
| **Lymphovascular Invasion (%)** | | | | | | | | |
| Negative | - | - | | - | 5 (55.6) | 4 (13.8) | - | 9 (23.7) |
| Positive | - | - | | - | 3 (33.3) | 21 (72.4) | - | 24 (63.2) |
| Biopsy only | - | - | | - | - | 3 (10.3) | - | 3 (7.9) |
| Missing | - | - | | - | 1 (11.1) | 1 (3.4) | - | 2 (5.3) |
| **Distal Recurrence (%)** | | | | | | | | |
| No | - | - | | - | 7 (77.8) | 20 (69) | - | 27 (71.1) |
| Yes | - | - | | - | 2 (22.2) | 9 (31) | - | 11 (28.9) |
| **Local Recurrence (%)** | | | | | | | | |
| No | - | - | | - | 6 (66.7) | 10 (34.5) | - | 16 (42.1) |
| Yes | - | - | | - | 3 (33.3) | 19 (65.5) | - | 22 (57.9) |
| **Disease Progression (%)** | | | | | | | | |
| No | - | - | | - | 3 (33.3) | 12 (41.4) | - | 15 (39.5) |
| Yes | - | - | | - | 6 (66.7) | 17 (58.6) | - | 23 (60.5) |
| **Mortality (%)** | | | | | | | | |
| Alive | All alive | All alive | | 6 (85.7) | 4 (44.4) | 11 (37.9) | - | 21 (55.3) |
| Dead | - | - | | 1***** (14.3) | 5 (55.6) | 18 (62.1) | - | 24 (44.7) |
