# Supplementary material for: MicroRNAs Targeting Oncogenes Are Down-Regulated in Pancreatic Malignant Transformation from Benign Tumors
Source: PLoS One. 2012 Feb 22;7(2):e32068. doi: 10.1371/journal.pone.0032068 (PMC3284550; doi:10.1371/journal.pone.0032068)
Supplement: Table S2 — Sequences of all primers used for KRAS luciferase plasmid construction. Red ends indicate sequences appropriate for the Mlu1 and HindIII restriction enzymes. Yellow highlighted areas indicate mutated nucleotides. (DOC) [file pone.0032068.s006.doc]

| **KRAS_A_WT** | Sense | CGCGCAGCGACAGTAGGATTTTTCAAACCTGGTATGAATAGAGC |
| --- | --- | --- |
|  | Antisense | AGCTGCTCTATTCATACCAGGTTTGAAAAATCCTACTGTCGCTG |
| **KRAS_A_MUT** | Sense | CGCGCAGCGACAGTAGGATTTTTCAAACCTGGATAGAATAGAGC |
|  | Antisense | AGCTGCTCTATTCTATCCAGGTTTGAAAAATCCTACTGTCGCTG |
| **KRAS_B_WT** | Sense | CGCGCGTGCTTTCTTTTGTGGGACATATGCAGTGTGATCCAGGC |
|  | Antisense | AGCTGCCTGGATCACACTGCATATGTCCCACAAAAGAAAGCACG |
| **KRAS_B_MUT** | Sense | CGCGCGTGCTTTCTTTTGTGGGACATATGCAGACAGATCCAGGC |
|  | Antisense | AGCTGCCTGGATCTGTCTGCATATGTCCCACAAAAGAAAGCACG |
